# Case report: Anterior midline decompression of a cervical epidural abscess: Technical note and case series of seven patients

**DOI:** 10.3389/fsurg.2022.988565

**Published:** 2022-12-26

**Authors:** Ahmed Zian, Mark P. Arts, Niels A. van der Gaag

**Affiliations:** ^1^ Department of Neurosurgery, Haaglanden Medical Center, The Hague, Netherlands; ^2^ Department of Neurosurgery, Leiden University Medical Center (LUMC), Leiden, Netherlands

**Keywords:** anterior decompression, surgical treatment, spinal epidural abscess (SEA), spinal infection, epidural abcess, anterior approach, infection, cervical spinal cord compression

## Abstract

**Background:**

A spinal epidural abscess (SEA) of the cervical spine is a relatively rare disease and is generally characterized by progressive neurological deterioration due to compression of the spinal cord. Up to 40% of cervical SEAs are located ventrally of the spinal cord. Urgent surgical intervention is warranted to decompress the spinal cord and collect material for cultures to guide antibiotic treatment. However, the optimal timing of the intervention is unclear, and the associated risk of spinal instability in the presence of an extensive infection is a significant clinical dilemma.

**Methods:**

In this paper, we present a novel surgical technique to treat a cervical SEA by anterior decompression through a linear transvertebral midline approach. This technique has the advantage of effectively draining the ventrally located SEA and obtaining material for bacteria culture while maintaining spinal stability without additional instrumentation.

**Results:**

This case study presents seven patients with cervical SEAs who were successfully treated with surgical decompression by this transvertebral linear midline technique and antibiotic treatment.

**Conclusion:**

Anterior decompression through a linear transvertebral midline approach for a ventrally located cervical SEA is a safe and pragmatic surgical procedure to achieve spinal cord decompression and collect bacteria culture without destabilizing the cervical spine.

## Introduction

A spinal epidural abscess (SEA) is a rare disease with an estimated incidence of 2–25 cases per 100,000 hospital admissions, of which 19%–26% are located in the cervical spine (1, 2). Ventral localization of cervical SEAs is reported in 37.2% of cases, and dorsal and circumferential localization in 32.6% and 30.2%, respectively (3). A cervical SEA is associated with progressive neurological deterioration due to compression of the spinal cord, leading to an inadequate vascular blood supply with consequent ischemic changes. The majority of patients present with some kind of neurological impairment (4, 5). Most of the infections are related to hematogenous or lymphatic spread from remote sources such as dermal, dentogenic, or urinary tract infections or endocarditis (2, 6, 7, 9, 10). Predisposing risk factors are diabetes mellitus, obesity, use of immunosuppressive medication, epidural anesthesia, and intravenous drug abuse (2, 11, 12). Regarding the causative organisms, a diverse range of micro-organisms have been described, although *Staphylococcus aureus* is the most prevalent (2).

An SEA should be treated with targeted systemic antibiotics based on bacterial culture with or without decompressive surgery, although the best strategy is not known (8, 13–16). In cases presenting with short-lasting neurological deterioration, i.e., within 24–36 h, and radiological findings of spinal cord compression, surgical decompression is the preferred initial treatment. With this strategy, it is possible to achieve the immediate reduction of spinal cord compression and to obtain material for culture tests to guide antibiotic treatment (10). Depending on the extent and localization of the SEA, various surgical approaches have been described to remove abscess tissue: anterior discectomy, corpectomy, micro-surgical aspiration by catheter drainage, and posterior approaches such as laminectomy or microscopic decompression (11, 13, 17, 18). In case of concomitant instability, spinal malalignment, or vertebral body collapse, there is a tendency for additional instrumentation, although literature is controversial as to whether this should be performed (22–24). The arguments in favor of instrumentation are biomechanical support to maintain spinal alignment and prevent kyphosis or vertebral collapse. An important argument against instrumentation refers to the risk of implant infection with associated nonunion and subsidence. Therefore, the oblique corpectomy technique without instrumentation has been described as an approach to drain ventral cervical SEAs (19, 20). In the present case series, we describe an alternative surgical technique for a ventrally located cervical SEA by a transvertebral midline decompression. The surgical procedure and outcome of seven patients is described.

## Methods

### Study design and study population

The design of the present study adopts a retrospective clinical case series. All patients with cervical SEAs who were treated with anterior decompression between 2013 and 2021 at the Haaglanden Medical Center and the Leiden University Medical Center were included in this case series. This study reviewed patients’ medical records, surgical notes, and photo documentation taken during their hospital stay along with all clinical follow-up notes.

### Surgical technique

**Figure 1:**
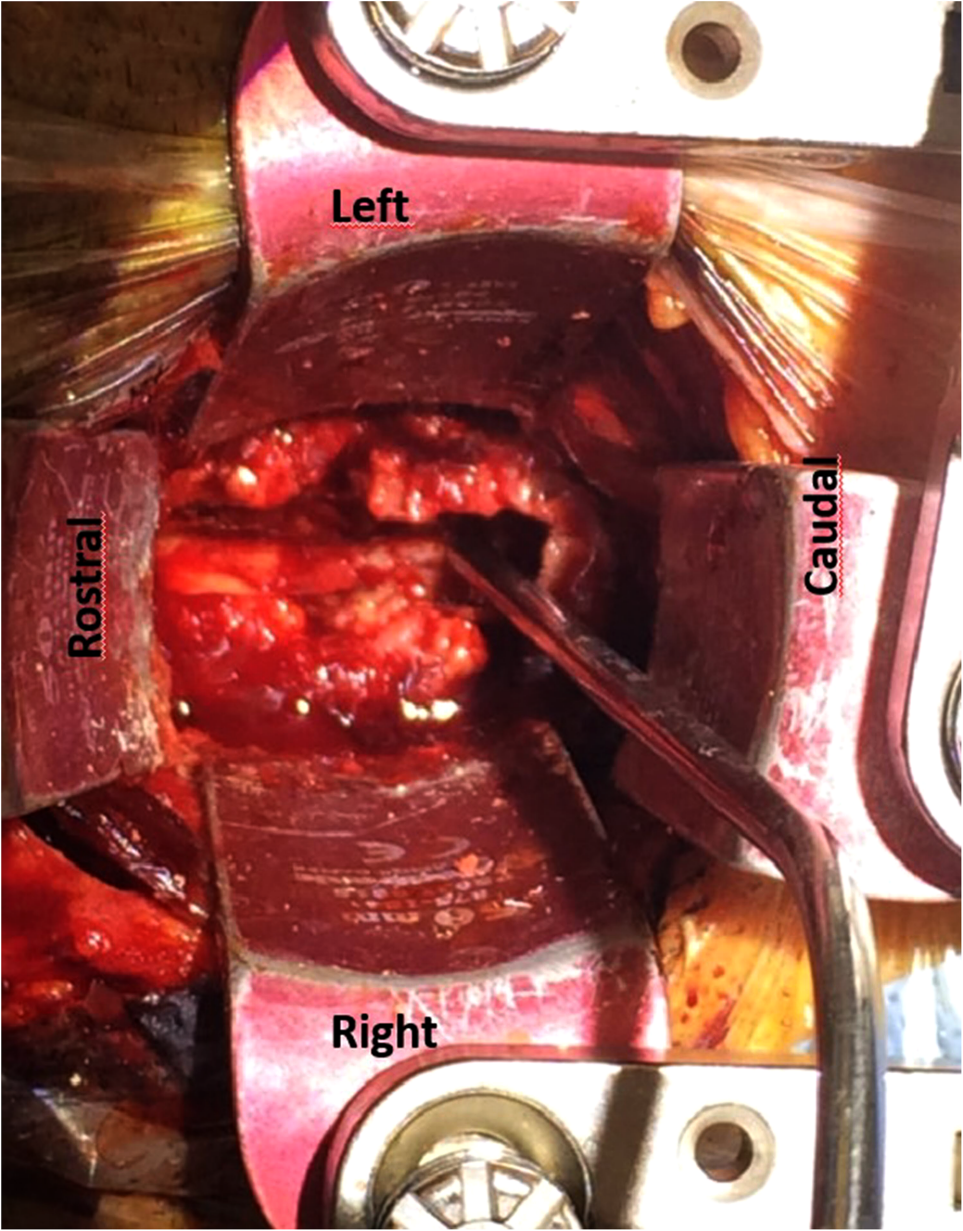
Operative image of case 4: an anterior view of the cervical spine with an anterior transvertebral decompression in the midline of vertebral bodies and intervertebral disc C4–C7. Through the trench the epidural space is reached with the dura mater as posterior border.

In the case of a non-septic patient, no prophylactic antibiotics were given perioperatively in order to minimalize the risk of a false-negative bacterial culture. In case of an already-initiated antibiotic treatment, this treatment was continued. In accordance with the classical anterior cervical discectomy procedure, the patients were placed in the supine position with the head in a neutral position and slightly extended. A horizontal skin incision was made at the affected cervical level, verified by a lateral radiographic examination. After exposure of the affected cervical segment(s), a vertical, linear midline trench was created up to the posterior longitudinal ligament with a large-diameter (5–7 mm), high-speed surgical drill. The trench could be easily extended over multiple vertebra and discs in the craniocaudal direction, based on extension as apparent on the preoperative MRI (Figure 1). The posterior longitudinal ligament is opened in the craniocaudal direction by a 1–2 mm Kerrison rongeur, and with extensive irrigation, abscess evacuation can be achieved without compromising the cervical integrity and stability. An intraoperative microscope was not used. No intervertebral cage or plate was implanted. At closure, a silicone vacuum drain was placed with the distal end placed under the inner layer of the deep fascia (prevertebral space) connected to a disposable drainage bag with an anti-backflow valve. An immobilizing rigid collar was not routinely applied. The drain was removed on postoperative day 1 or 2. Broad-spectrum systemic antibiotic treatment was started directly after the abscess evacuation to increase the likelihood of an accurate diagnosis of infection. Narrowing the broad-spectrum antibiotic and the duration of treatment was based on the sample culture of the abscess, in close consultation with the microbiologist or infectious disease specialist. A follow-up MRI scan was performed to assess the possible remaining spinal cord compression and to evaluate the effect of antibiotic treatment.

#### Clinical and radiological follow-up

**Figure 2:**
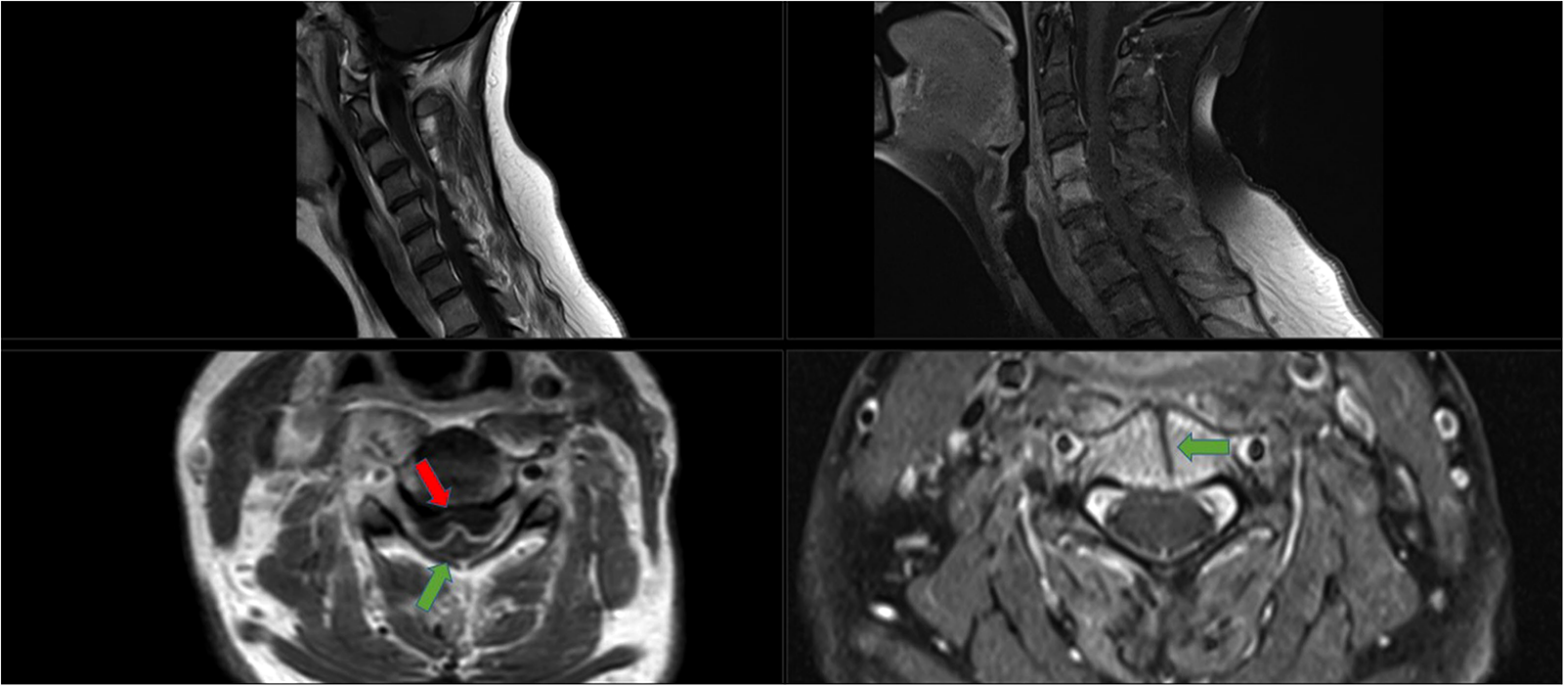
Radiological images of case 3. Left images: Pre-operative MRI of the cervical spine (T1-weighted images post-gadolinium infusion, slice thickness 4.0 mm). Upper image shows a sagittal view with a ventral cervical epidural abscess on C4–5 with compression of the spinal cord. Lower image shows the axial view, red arrow points to the abscess, green arrow the spinal cord. In between a hyper-intense contrast signal indicating the existence of an abscess capsule. Right images: Post-operative MRI of the cervical spine, 2 weeks post-operative and antibiotic treatment with cefuroxime (T1-weighted images post-gadolinium infusion, slice thickness 3.0 mm). Upper image: Sagittal view with a hyper-intense signal of the vertebral bodies of C4 and C5, but no abscess. Furthermore, the cervical spine is normal alignment; there is no sign of kyphosis or collapse of the vertebral bodies. Lower image: axial view with the green arrow pointing to the anterior transvertebral trench and the spinal cord after adequate decompression and antibiotic treatment.

**Figure 3:**
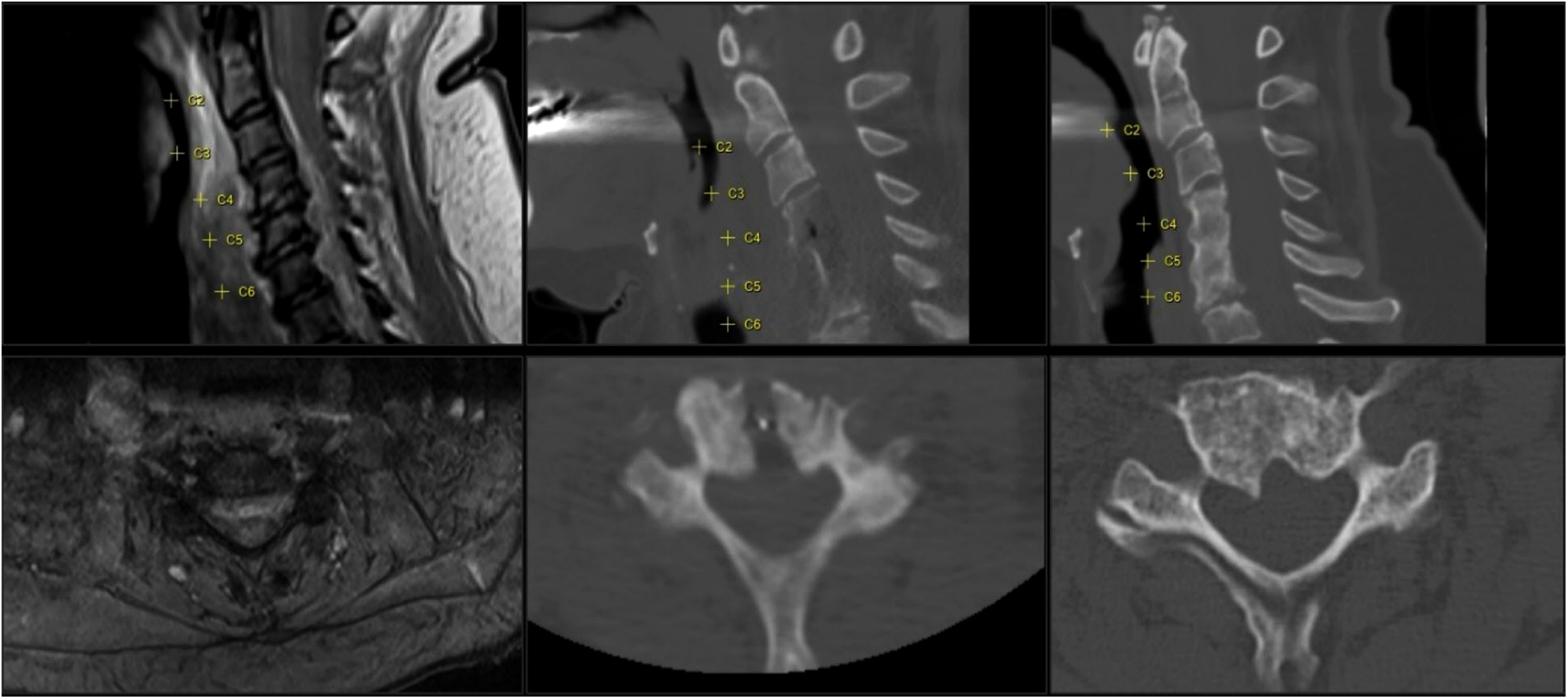
Imaging of case 2 with a cervical abscess extending from C2 to C7. Left images show the pre-operative MRI sagittal and axial view (T2 with gadolinium). Middle images show the postoperative CT (CT bone view, sagittal and axial view); the anterior transvertebral midline decompression is viewed from C4 to C6. Axial view shows corpus C5. Right images show a follow-up CT at 4 months (CT, bone view, sagittal and axial view); ossification of the gutter can be seen and vertebral fusion of C4–6.

In our opinion, the postoperative radiological follow-up should exist of a cervical x-ray (lateral and anteroposterior view) with the patient in a sitting or standing position to assess the cervical spinal alignment with an axial load as soon as the patient is able to mobilize. In case the spinal stability is doubtful, a CT scan can be performed on the cervical spine. In order to evaluate the extent of the abscess evacuation, an MRI scan can be repeated 3 days after surgery, or earlier if indicated, in case of further postoperative neurological deterioration. In case of positive microbial tissue culture, adequate antibiotic therapy, and no further neurological deterioration, an MRI scan should be repeated after 6 weeks to evaluate the residue of the abscess followed by an adjustment of the antibiotic therapy or its discontinuation. In case of further neurological deterioration in an earlier phase, an MRI scan should be repeated earlier to evaluate the progression of the abscess (Figure 2). Three months after surgery, a CT scan is recommended for the assessment of bone growth and fusion of the trench through the vertebral body. In case of a good recovery without signs of spinal instability, the patient can be discharged (Figure 3).

#### Informed consent

As this was a retrospective study, no informed consent was retrieved.

## Results

Between January 2013 and January 2021, seven patients were identified with a cervical SEA and underwent surgical treatment in Haaglanden Medical Center and Leiden University Medical Centre. Five patients (71%) were male, and the age at surgery was in the range of 37–63 years (median 59 years). The duration between the initial symptoms and surgery was in the range of 1–21 days (median 11 days). At the time of hospital admission, all patients presented with fever, elevated serum C-reactive protein, and neurological deterioration, ranging between American Spinal Injury Association (ASIA) Impairment Scale A and C. In four patients, antibiotic treatment was administered for a different cause before the diagnosis of a cervical SEA was established. In five cases, the SEA was located only in the cervical spine, and two cases presented with an SEA in the thoracic and lumbar spine as well (Table 1).

**Table 1:** Demographic data, comorbidities, duration of symptoms in days, affected cervical levels and co-affected spinal regions.

Case (year of presentation)	Sex	Risk factors	Clinical presentation	Duration of symptoms in days prior to surgery	Pre-operative antibiotic management	Affected cervical levels	Other spinal segments affected
1 (2013)	Male	Septic, m. psoas abscess (*Staphylococcus aureus*)	Septic shock, sedated and intubated	11	None	C3–4	None
2 (2014)	Female	Hypertension	Progressive proximal paraparesis	1	None	C2–7	L5–S1
3 (2015)	Male	Alcoholic hepatitis	Paresis UE grade 3–4	14	Cefuroxime/gentamycine	C4–5	C3–S1
4 (2015)	Male	Diabetes mellitus, old bullet injury leg, recent dental injury	Neck pain	14	None	C5–6	None
5 (2019)	Male	UTI (*Escherichia coli*)	Neck pain, progressive weakness UE	21	Ceftriaxone/metronidazole after CT-guided punction	C7–T1	None
6 (2020)	Female	Recent compartment syndrome arm needing fasciotomy, UTI (*Klebsiella pneumoniae*), bacteremia (*S. aureus*)	Neck pain, fast motor function loss UE and LE	10	Cefuroxime	C2–5	None
7 (2021)	Male	Prostatitis (*E. coli*)	Fall followed by right-sided hemiparesis grade 3	1	Cefuroxime	C4–6	None

Demographic data of 7 cases treated between 2013 and 2021 in The Hague, the Netherlands. Symptoms (UE, upper extremities; LE, lower extremities) and pre-operative duration are showed.

Six cases underwent an anterior decompression through a linear transvertebral midline approach without instrumentation (Table 2). In one case, additional instrumentation was implanted; a laminectomy with a posterior fixation in a secondary procedure was needed due to spondylitis with progressive kyphosis after the primary anterior transvertebral midline decompression. This was the only case that was treated with a stiff neck collar for additional anterior support after posterior fixation. In three cases, increased neurological deterioration was observed directly postoperatively, both an increase of one grade of the ASIA Impairment Scale. At the follow-up, a recovery of 1–2 scales was observed. There were no further complications observed during the postoperative course. Microbial cultures revealed a positive identification of the causative agent in all cases (Table 2). Patients were treated with antibiotic therapy after surgery for at least 6 weeks intravenously; three cases received flucloxacillin, two cases received ceftriaxone, and two cases received cefuroxime. Four cases received prolonged antibiotic therapy orally, ranging between 1 and 9 months.

**Table 2:** Overview of surgical and antibiotic management, peri-procedural neurological status.

Case	Surgical management	Pre-op neurological status (ASIA)	24 h post-op (ASIA)	Follow-up post-op (months)	Impairment at follow-up (ASIA)	Imaging at follow-up	Organism	Antibiotic treatment	Stiff collar immobilization
1	Anterior transvertebral midline decompression C3–4	Tetraparesis gr 3 (C)	B	3	D	MRI + CT	*S. aureus*	6 weeks flucloxacillin iv	–
2	Anterior transvertebral midline decompression C4–6	Proximal paraparesis gr 3 (D)	C	12	D	MRI + CT	*S. Areus*	6 weeks flucloxacillin iv, 9 months orally	–
3	Anterior transvertebral midline decompression C4–5	UE paresis gr 3 (D)	D	8	E	MRI	*S. aureus*	6 weeks flucloxacillin + ceftriaxone, 4 months clindamycin	–
4	Anterior transvertebral midline decompression C4–7	UE paresis gr 3 (D)	D	N/A	N/A	N/A	*Rothia mucilaginosa*	6 weeks flucloxacillin iv	–
5	Primary anterior transvertebral midline decompression C7–T1, followed by a secondary laminectomy C4–Th1 with posterior spondylodesis C3–T2	Posterior cord syndrome, maximal motor function (D)	C	12	D	CT (1, 6 and 12 months)	*E. coli*	6 weeks ceftriaxone, 4 months amoxicillin	For 6 months post-op
6	Anterior transvertebral midline decompression C4–5	Tetraplegia (A)	A	2	D	MRI	*S. aureus*	6 weeks cefuroxime	–
7	Anterior transvertebral midline decompression C4–6	Right-sided hemiparesis gr 2–3 (C)	D	1	D	MRI	*Escherichia coli*	6 weeks cefuroxime + 4 weeks cotrimoxazole	–

Treatment per patient. Neurological and treatment status: pre-operatively, 24 hours post-operatively, and at follow-up. Neurological status is described in ASIA (American Spinal Injury Association) Impairment Scale and a resume of the neurological examination expressed in the MRC-grade (Muscle Power Assessment) for the upper extremities (UE) and lower extremities (LE); A: no sensory/motor function below neurological level, B: sensory function, but no motor function below neurological level, C: less than grade 3 motor function below neurological level, D: grade 3 or more below neurological level, E: neurologically intact.

In one case, the follow-up was missing due to the patient being of foreign origin and returning to his home country. For the six other cases, the follow-up varied between 1 and 12 months (median 4 months). During the follow-up, six cases showed neurological improvement, while the condition of one patient remained unchanged.

## Discussion

A cervical SEA implies a severe disease that is associated with extensive treatment and a potentially detrimental outcome. In this series, we present seven cases with a cervical SEA, effectively treated with an anterior and linear decompression in the midline in order to evacuate a ventrally located cervical SEA. Additional instrumentation was required for only 1 case due to accompanying spondylitis and progressive kyphosis. At the latest follow-up, 6 (86%) cases reached a clinically stable or improved neurological situation, and no further deterioration or signs of spinal instability were observed.

In case of a ventrally located SEA, multiple approaches are described, such as anterior discectomy with or without fusion, corpectomies, micro-surgical aspiration by catheter drainage, and posterior approaches followed by antibiotic treatment. In our opinion, posterior approaches for ventral abscesses are not preferred while an anterior approach results in the direct exposure of the target area. Furthermore, an anterior, linear midline decompression provides access to the abscess and maintains spinal integrity and stability without necessary implants. Although the use of titanium cages in infected tissue is assumed to be safe, discectomies and corpectomies can be avoided as our less-invasive approach can lead to the same result without the additional risk of implant-related failure or infection (23). Existing studies on the treatment of cervical SEAs from a surgical point of view are summarized in Table 3. Most surgical cases are treated rapidly after clinical presentation, preferably in an early stage, to prevent further neurological deterioration. The oblique corpectomy, according to Bernard George, is an equivalent approach to our technique but with an anterolateral rather than midline entry (19). Kunert et al. present the results of four cases with an oblique approach with a comparable outcome to our series (20). In comparison with the oblique approach, the midline approach seems to be anatomically more pragmatic as the vertebral midline itself is an easy landmark. In addition, a linear midline approach leads to two symmetrical columns of the vertebral body and a symmetrical distribution of the axial load. Lastly, the midline approach prevents potential injury to the radices as neuro-anatomically only the dura mater must be respected as the posterior border.

**Table 3:** Overview of literature with reports of surgical treatment of cases with ventral cervical SEA.

Author	Cases	Surgical	Immediate decompression	Ventral abscess	ACDF	Corpectomy	Combined	Other	Neurological stable/improvement (%)	Complications (%)
Rosell, 1998 (18)	1	1	–	1	–	–	–	Endoscopic	100	–
Muzii, 2006 (17)	8	8	8	8	8	–	–	–	100	–
Shousha, 2021 (21)	30	30	30	2	2	2	–	–	90	10
Ghobrial, 2015 (5)	40	40	40	12	4	4	26	–	>40	6
Alton, 2015 (4)	62	62	38	13	14	10	5	–	100	–
Kunert, 2016 (20)	4	4	4	4	–	–	–	OC	75	–
Shweikeh, 2017 (13)	16	16	–	8	5	1	2	–	–	25

Seven articles consisting of retrospective reviews and case reports. Results are selected based on surgical approaches for ventral cervical SEA. SEA, spinal epidural abscess; OC, oblique corpectomy.

The current mainstay treatment of a cervical SEA generally consists of antibiotic treatment; surgical decompression is reserved in case of neurological deterioration due to spinal cord compression. However, a study by Alton et al. presents an outcome in favor of early surgical decompression in comparison to an initial medical treatment only (4). Compared with patients treated medically or with delayed surgery after failed therapy, patients undergoing early surgery within 24 hours showed a significant improvement in their motor scores (MS) and no decline in MS, according to the ASIA (0–100 points). This was based on the MS assessment after completion of the 6–12 weeks of postoperative antibiotic treatment. The mean MS difference was 2.3 ± 4.4 points for the medical group, −15.9 ± 24.9 points for the delayed surgery group, and 11.9 ± 19.5 points for the early surgery group. These results concur with our point of view that early surgery can stabilize further neurological deterioration. Furthermore, we consider an atraumatic evacuation more feasible in an early stage of the disease when an abscess is more likely to be fluid of consistency.

In the process of our clinical work, the choice of antibiotic is determined by the microbiologist and/or an infectious disease specialist. For every patient, a customized antibiotic treatment is determined based on the infectious information available. In case of an infection of unknown origin, a broad-spectrum regimen is started consisting of ceftriaxone and metronidazole, according to the Dutch national guideline of the National Workgroup of Antibiotic Treatment (SWAB) on the treatment of epidural abscesses of the central nervous system (25). In our case series, two cases showed an *Escherichia coli* infection, which needed different antibiotic treatment from the cases with a *Staphylococcus aureus* infection. This was based on culture results and finally on antibiotic susceptibility testing.

In our series, we observed one case who needed a secondary surgery for additional posterior fixation to treat progressive cervical kyphosis due to spondylitis. No other complications were observed, and all cases showed successful radiological results at follow-up. With respect to neurological outcome, two cases in our series stabilized: the patient with a widespread spinal infection that also involved the thoracic and lumbar region and the patient who required the secondary surgery. All other cases improved by one or several ASIA grades, and one case even recovered from ASIA A preoperatively to ASIA D postoperatively in 2 months. Nevertheless, a full recovery to ASIA E was rare, which underlines the severity of the disease. These results agree with those of other studies, which also report a stabilization of functional deterioration and even some recovery. In three cases, a decrease of one ASIA scale was observed at the first postoperative follow-up; this could be explained by temporary deterioration due to epidural rinsing and the possible manipulation of vulnerable infected neuronal tissue. Regarding the complication risk of postoperative irreversible neurological deterioration or death, earlier studies described a complication risk of 6%–25% (Table 3). In our case series of seven cases, six cases were assessed at follow-up and showed no further deterioration or improved. One case was lost at follow-up. Of these six cases, all survived at the follow-up ranging between 1 and 12 months. With these results of the anterior midline decompression of a cervical epidural abscess, we would like to introduce this as a recommended approach for the evacuation of a cervical ventral epidural abscess.

## Conclusion

Anterior decompression through a linear transvertebral midline approach for a ventrally located cervical SEA is a safe and pragmatic surgical procedure to achieve spinal cord decompression and collect bacteria culture without destabilizing the cervical spine. Together with subsequent antibiotic treatment based on the causative agent, this surgical approach leads to an adequate and curative treatment of this fulminant and disabling spinal infection. An adequately timed radiological follow-up by x-ray, CT, and MRI contributes to safe postoperative management.

## Data Availability

The original contributions presented in the study are included in the article/Supplementary Material, further inquiries can be directed to the corresponding author.
